# Evaluation of pasture herbage intake equations based on dairy cow behavior recorded with the RumiWatch system

**DOI:** 10.3168/jdsc.2025-0752

**Published:** 2025-05-22

**Authors:** Fredy Schori, Thorsten Haak, Jessica Werner

**Affiliations:** 1 Ruminant Nutrition and Emissions, Agroscope, Posieux, 1725, Switzerland; 2 Institute of Animal Science, University of Bonn, Bonn, 53115, Germany; 3 Institute of Agricultural Sciences in the Tropics, University of Hohenheim, Stuttgart, 70599, Germany

## Abstract

Accurate estimation of individual feed intake is essential for calculating feed efficiency, planning diets, monitoring cow herds, and managing grazing cows. This study aimed to evaluate the performance and applicability of estimation equations developed to predict pasture herbage DMI (PHDMI) in dairy cows using behavioral traits recorded and scored by the RumiWatch system. The study had 4 primary objectives: (1) to compare the behavioral characteristic outputs of 2 versions of the RumiWatch converter (0.7.3.31 and 0.7.3.36); (2) to validate existing PHDMI estimation equations using an independent dataset of Swiss Fleckvieh and Holstein cows; (3) to assess the suitability of the equations for estimating the intake of fresh herbage and hay fed indoors; and (4) to determine whether the RumiWatch halter impedes feed intake. Nine behavioral characteristics relevant to the PHDMI estimation equations were evaluated with both converters and found to be consistent, with negligible differences in the results. The validation of 6 of the PHDMI estimation equations appeared to provide individual intake predictions for grazing cows with low mean bias and may be suitable for herd-level assessments. For individual cow PHDMI estimation, the utility appeared limited, as only a very small proportion of PHDMI variability was explained by the equations. However, the calculated root mean square error of ~15% is in the acceptable range, according to the literature. As expected, the equations largely underestimated the intake of fresh forage and hay fed indoors, negating their suitability for estimating the intake of combinations of grazed herbage and barn-fed forages. A comparison of hay intake in cows with or without the halter showed no evidence that wearing a correctly fitted RumiWatch halter reduced hay intake. These results highlight the potential of the RumiWatch system and the associated estimation equations to support herd-level PHDMI and underscore the need for further testing to improve its performance in diverse feeding environments.

## Highlights

• When properly fitted, the RumiWatch halter does not affect feed intake.
• Both converters yield similar results, transcoding the halter records consistently.
• The equations are suitable for estimating grazing cow intake at the herd level.
• The equations cannot estimate fresh herbage intake in barn conditions.
• The equations cannot estimate hay intake.

## Graphical Abstract

**Figure:**
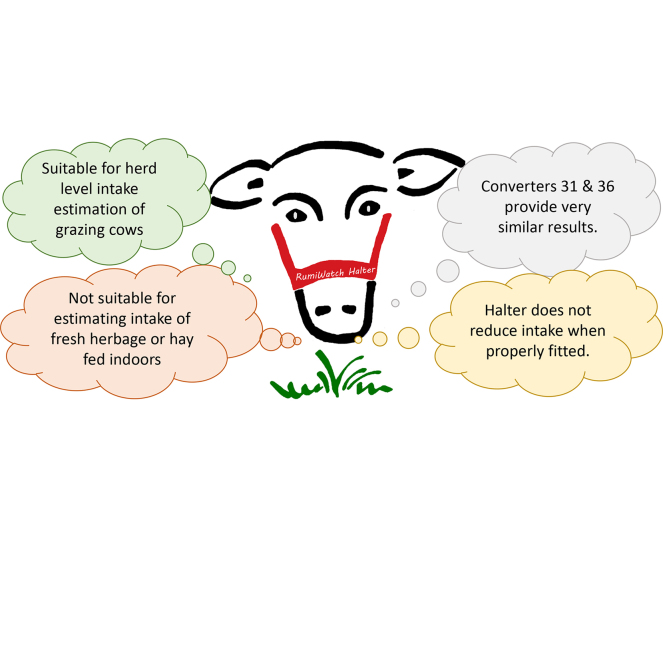
**Summary:** This study aimed to evaluate the performance and applicability of equations developed to predict pasture herbage dry matter intake in dairy cows using behavioral traits as recorded by the RumiWatch system. The results highlight the potential of the RumiWatch system and the associated equations to estimate pasture herbage dry matter intake at the herd level under full-day grazing conditions. However, they also underscore the need for further testing to improve the performance of the prediction equations in diverse feeding environments.

Individual feed intake is a key variable in calculating feed efficiency and is expected to play an important role in future dairy cow selection. This measure is also crucial for feed planning, herd monitoring, and pasture management (Dillon, 2006; Seymour et al., 2019). Experimental methods used to determine individual pasture herbage DMI (**PHDMI**), such as marker methods, are only suitable for a limited number of dairy cows. Moreover, these methods are costly and labor intensive and require varying degrees of chemical analysis. The RumiWatch behavioral recording system (ITIN+HOCH GmbH, Liestal, Switzerland) aims to offer a method for estimating the daily PHDMI of individual dairy cows. The system was previously evaluated among grazing dairy cows by Rombach et al. (2018) using the converter 0.7.3.2 and the improved converter 0.7.3.11. The RumiWatch converters use different algorithms to transcode the recorded signals from the halter, such as acceleration in 3 directions and pressure differences, into eating and rumination behavioral characteristics. Rombach et al. (2023) validated RumiWatch converter 0.7.3.31 (**Con31**), which attempts to distinguish between prehension bites and mastication chews during grazing, and Werner et al. (2018) and Norbu et al. (2021) validated the further refined RumiWatch converter 0.7.3.36 (**Con36**). Estimation equations for the individual PHDMI of dairy cows were derived based on a wide range of predictors (Rombach et al., 2019). In addition, estimation equations for individual PHDMI were developed using dairy cows' eating and rumination behavioral characteristics reported by the RumiWatch system as exclusive predictors (Schori et al., 2020). To attempt to automate the PHDMI estimation based on the behavioral characteristics recorded by the RumiWatch system, further clarification is required. (1) Because the PHDMI estimation equations of Rombach et al. (2019) and Schori et al. (2020) were developed using behavioral characteristics obtained with Con31, it is necessary to determine whether Con31 and Con36 produce consistent and comparable results. To the authors' knowledge, this comparison between the results of the 2 converters has not yet been published. The hypothesis is that there are no relevant differences in behavioral characteristics when the recorded signals are evaluated with Con31 or Con36. This is based on the manufacturer's information that Con36 is based on Con31 and provides additional behavioral characteristics. (2) To test the robustness of the estimation equations, some of the PHDMI equations of Rombach et al. (2019) and Schori et al. (2020) are planned to be validated with more recent data from an independent study on grazing Swiss Fleckvieh and Holstein dairy cows (Haak et al., 2024). The target would be to have an estimation error for the individual PHDMI estimate of less than 20%, which, according to Fuentes-Pila et al. (1996), would indicate acceptable accuracy of the estimates. (3) The term “pasture-based feeding systems” encompasses a wide range of approaches, including the proportion of grazed herbage included in the daily diet of dairy cows (Morales et al., 2024). Therefore, the intention was to examine the performance of the estimation equation for PHDMI with fresh cut herbage and hay fed indoors, as these forages are used as supplements to grazed herbage (Ineichen et al., 2016). A previous experiment (Dohme-Meier et al., 2014) demonstrated that for a given herbage quality, the feed intake rate of dairy cows fed herbage indoors is significantly higher than when grazing on pasture. Consequently, estimates of the fresh herbage intake of dairy cows fed indoors based on the PHDMI equations are predisposed to contain a large mean bias. Regarding hay rations, there is little concrete evidence of the performance of PHDMI equations. (4) Another important aspect that, to the best of the authors' knowledge, has not been studied or published is whether the correctly fitted RumiWatch halter impedes the feed intake of dairy cows. It may be hypothesized that when the RumiWatch halter is applied as recommended (Rombach et al., 2018), the animals are not hindered in their feed intake.

The dataset from Haak et al. (2024) was used to compare the 2 converters and to validate the PHDMI estimation equations. It includes 4 feeding experiments carried out in Switzerland. Three of these were grazing experiments with Holstein and Swiss Fleckvieh cows, all conducted on the organic farm Ferme École de Sorens (Sorens, Switzerland). The fourth was an indoor feeding experiment at the Agroscope experimental farm in Posieux, Switzerland, where Holstein cows were fed fresh herbage. In the grazing experiments, cows had access to pasture for 16 to 19 h per day. Across all 4 experiments, the cows received only small amounts of supplementary feed (on average between 0.3 and 1.0 kg DM of concentrate per cow per day, depending on the experiment). Average daily milk yield per experiment ranged from 16.0 to 22.6 kg per cow, and cows were, on average, between 122 and 233 DIM, depending on the experiment. Reference PHDMI values were determined using the double marker method with *n*-alkanes over a period of 7 d. When fresh herbage was fed indoors, feed intake was additionally measured using weighing troughs (Isentec RIC system, Hokofarm Group, Emmeloord, the Netherlands). The feeding and rumination behavior of all cows was recorded using the RumiWatch system. Further information on the experimental conditions, ethical statement, and reference methods for DMI determination are provided in Haak et al. (2024).

To compare the 2 converters, the RumiWatch halter recordings of day and night grazing dairy cows and those fed exclusively fresh herbage in the barn were evaluated with Con31 and Con36. In the evaluation by Rombach et al. (2023), Con31 showed the following mean absolute deviation percentages compared with visual observations: 6.0% for eating time, 11.2% for eating chews, 12.4% for prehension bites, 2.1% for rumination time, and 3.9% for rumination chews. Observations were included in our analysis if plausible RumiWatch recordings were available for 6 d or more per measurement period. The 1-h summaries converted with Con36 were summed to 24-h summaries. Total eating time, rumination time, rate of rumination chews, total eating chews, and total other chews were calculated as the mean of the 24-h summaries for the 7-d measurement period. The behavioral traits “eating time head up and down,” “other chews (at pasture),” and “chews per bolus” made during the cow's stay in the barn (5–7 h and 12–18 h or 16–18 h depending on the recording period) were not included in the evaluation. Finally, 72 out of 84 observations, consisting of the daily PHDMI averaged over 7 d and the average daily behavioral traits over at least 6 d of the measurement period, were considered for the evaluation of the 6 PHDMI estimation equations. The WSB3 estimation equation, proposed by Rombach et al. (2019), is composed as follows: PHDMI (kg/d) = 13.728 − 3.1564 × lactose (%) + 0.0096 × BW (kg) − 0.0003 × total eating chews (n/d) + 0.0274 × total eating time at pasture − 0.0006 × total eating chews head down + 0.0008 × eating chews head down at pasture. Estimation equations S1 to S5 were published in Schori et al. (2020), including information on intercepts and regression coefficients.

To evaluate the prediction equations with fresh herbage fed indoors, the reference intake was determined using the *n*-alkane double marker method. The aim was to use the same reference method for the evaluation of pasture intake. The difference between the intake measured with the weighing troughs (Isentec RIC system, Hokofarm Group, Emmelroord, the Netherlands) and the *n*-alkane double marker method was 0.47 kg DM/cow per day, 2.3% less on average for all 16 dairy cows in the study. This result shows that the *n*-alkane double marker method as applied by Haak et al. (2024) works well, at least for dairy cows fed fresh herbage indoors.

The suitability of the PHDMI prediction equations for the DMI of hay was tested using new experimental data not included in the Haak et al. (2024) dataset. All experimental procedures for this hay experiment were in accordance with the Swiss Animal Welfare Guidelines and were approved (no. 2022-30-FR) by the Animal Care Committee of the Canton of Fribourg, Switzerland. The 16 Holstein and Red Holstein cows used in this experiment were housed in the same freestall barn and had an average of 3.1 lactations (±2.0 SD) and 261 (±27 SD) DIM at the beginning of the first week of measurement. For 4 wk, the dairy cows in this experiment were fed a hay-only diet supplemented with minerals only. The mean and SD of the energy and nutrient contents of the 3 pooled hay samples from wk 2 to 4 were 901 ± 2.0 g DM/kg, and per kg DM: 247 ± 2.2 g ADF, 483 ± 5.5 g NDF, 132 ± 2.9 g CP, and 5.7 ± 0.02 MJ NEL. The reference intake was measured with the weighing feed troughs (Isentec RIC system) and corrected with the DM content of the hay. The first 2 wk were used to acclimate the experimental cows to the diet, and from the fourth day of the second week, the dairy cows wore RumiWatch halters for acclimation. On average, the cows produced 22.8 ± 4.4 (SD) kg of energy-corrected milk and weighed 687 ± 44 (SD) kg BW during the second adaptation week. During the first measurement week (wk 3), all 16 dairy cows wore the RumiWatch halter. During the second measurement week (wk 4), half of the dairy cows were removed from the RumiWatch halter to examine the influence of the RumiWatch halter on DMI.

The estimation equations were evaluated using R version 4.2.3 (R Core Team, 2024). The root mean square error (**RMSE**) was calculated using the “Metrics” package. Error allocation in the error of central tendency (**ECT**), error of regression (**ER**), and error of disturbance (**ED**) was calculated according to Tedeschi (2006; formula 20). The concordance correlation coefficient (**CCC**), local and scale shift, and bias correction factor were calculated using the “epiR” package. To compare the intake rates and hay DMI of different cows with and without the RumiWatch halter during the same measurement week, a Welch 2-sample *t*-test was performed using R. For hay DMI within the same cows, with and without the RumiWatch halter, a paired *t*-test was conducted.

In the converter comparison, only the behavioral characteristics relevant to PHDMI estimation were compared: eating time head up, head down and total, rumination time, eating chews head down, rumination chews, other chews, rate of rumination chews, and chews per rumination bolus (Table 1). Altogether, 630 daily records from 54 individual lactating dairy cows were analyzed. As hypothesized, the differences between the results of the behavioral traits analyzed with Con31 and Con36 are practically irrelevant, at least under the conditions tested. The largest percentage of RMSE was found for the characteristic “other chews” (0.83%), followed by “eating time head up” (0.22%), and all other traits showed values below 0.1%.

**Table 1:** Results of the comparison between the RumiWatch converters 0.7.3.31 (Con31) and 0.7.3.36 (Con36), based on a total of 630 daily records from 54 individual dairy cows

Trait	Mean^1^	SD^1^	Diff_Ø^2^	SD^2^	RMSE^3^ (%)
Eating time (min/d)					
Head down	480	111	0.011	0.248	0.05
Head up	138	72.4	−0.008	0.305	0.22
Total	618	80.6	0.003	0.503	0.08
Rumtime^4^ (min/d)	439	76.1	0.005	0.163	0.04
Chews (n/d)					
Eating head down	36,271	9,440	0.25	6.45	0.02
Rumination	27,377	5,751	0.321	8.49	0.03
Others	1,213	682	−0.051	10.1	0.83
Rumchews rate^5^ (n/min)	69.2	4.65	−0.00003	0.001	0.001
Chews per bolus (n/n)	54.7	7.08	−0.0002	0.009	0.02

1 Mean and SD of the Con31 evaluation.

2 Mean difference and SD of the difference between Con31 and Con36.

3 Root mean square error of Con36 based on Con31, in percent.

4 Rumination time.

5 Rate of rumination chews.

Table 2 shows the results of the evaluation of the 6 estimation equations using the PHDMI data from Haak et al. (2024). The average estimated PHDMI per equation was between 13.1 and 14.0 kg DM/cow per day, with an observed PHDMI of 13.9 kg (±1.61 SD) DM/cow per day. The SD of the estimated PHDMI was also close to the observed SD, ranging from 1.22 to 1.82 kg/cow per day. The r and CCC (r and CCC ranged from 0.06 to 0.30) were close to each other and were all positive but indicate a weak relationship between the observed and estimated PHDMI. According to R^2^, only a small part of the variability (maximum 9%) was explained. A more practical criterion is the RMSE, which, based on the observed intake, yielded a percentage error of 14% to 14.7%. According to Fuentes-Pila et al. (1996), an RMSE of less than 10% is considered a satisfactory intake prediction, whereas a value between 10% and 20% is deemed acceptable. The distribution of the error shows that the mean bias based on the ECT and local shift values for the estimation equations is very small. However, in this context, the estimation equation WSB3 is somewhat out of line. The bias correction factor also indicated a rather minimal model mean bias. The error sources ER and ED account for the largest part of the RMSE, with more than 99%. Unfortunately, PHDMI values in the range of 1 to 10 kg DM/cow per day are missing from the validation dataset. Additional values in this range may improve r, CCC, R^2^, and ER, which need to be clarified. The average PHDMI of a grazing dairy herd might be well estimated if the behavioral characteristics of a few focal animals are recorded and the conditions are similar to those in the test or validation dataset. Regarding the usefulness of individual dairy cow PHDMI estimates depends on what is to be done with them. For example, they may be sufficient to discriminate between highly efficient and inefficient dairy cows.

**Table 2:** Key metrics for evaluating behavioral models for estimating pasture herbage DM intake (PHDMI); values are kg/d unless otherwise noted or not required (n = 72, each value represents the average PHDMI over 7 consecutive days; overall mean = 13.9 kg/d, SD = 1.61)^1^

Item	WSB3	S1	S2	S3	S4	S5
Mean PHDMI	13.1	13.8	14.0	13.9	13.8	13.9
SD PHDMI	1.38	1.22	1.69	1.82	1.77	1.74
Pearson r	0.24	0.06	0.23	0.29	0.30	0.29
Coefficient of determination (R^2^)	0.06	0.003	0.05	0.09	0.09	0.08
RMSE^2^	2.01	1.95	2.05	2.04	1.99	1.99
ECT^3^ (%)	15.99	0.11	0.50	<0.01	0.19	0.04
ER^3^ (%)	24.1	33.0	41.6	43.6	41.1	40.8
ED^3^ (%)	59.9	66.9	57.9	56.4	58.7	59.2
CCC^4^	0.21	0.06	0.22	0.29	0.30	0.29
Local shift (μ)	−0.54	−0.05	0.09	0.007	−0.05	−0.02
Scale shift (ν)	0.86	0.76	1.05	1.13	1.10	1.08
Bias correction factor (C_b_)	0.86	0.96	0.99	0.99	0.99	1.00

1 WSB3, S1, S2, S3, S4, and S5 are the behavior-based estimation equations of PHDMI according to Rombach et al. (2019) and Schori et al. (2020).

2 Root mean square error.

3 Error of central tendency (ECT), error of regression (ER), and error of disturbance (ED) according to Tedeschi (2006).

4 Concordance correlation coefficient.

The proportion of grazed herbage in a dairy cow's daily ration can vary considerably (van den Pol-van Dasselaar et al., 2020) during the growing season. Among other factors, fresh herbage could also be fed indoors to dairy cows as a supplement to pasture, which was the reason for testing the estimation equation for fresh herbage intake in the barn. The observed DMI of fresh herbage in the barn averaged 19.8 (±1.88 SD) kg DM/cow per day for 15 cows and was considerably higher than the DMI of grazed herbage. One cow was excluded from the analysis because the behavioral records did not appear to be realistic related to the RumiWatch system or its application. As expected, the intake of fresh herbage in the barn was considerably underestimated by the equations developed to estimate PHDMI, as indicated by the mean estimated intake (11.9 kg DM/d with WSB3 to 16.0 kg DM/d with the S2 equation) and the local shift (−5.30 for WSB3 to −2.55 for S4 equation). Based on the results of Dohme-Meier et al. (2014), this was to be expected, as cows in the barn achieve a substantially higher DM intake rate with fresh herbage compared with a pasture environment. This is also reflected in the evaluation dataset (Haak et al., 2024) with a herbage intake rate of 23.9 (±3.38 SD) g DM/min at pasture versus 37.5 (±5.54 SD) g DM/min in the barn (*P* < 0.001).

The PHDMI prediction equations were also tested for the DMI of hay. This is because hay is also used as a forage supplement for grazing dairy cows and is fed indoors or offered directly through hay feeders at pasture. The observed DMI of hay in the barn averaged 20.7 (±2.09 SD) kg DM/cow per day for 16 cows during the first measurement period and was higher than the DMI of grazed herbage. Of the 24 cows wearing a RumiWatch halter during the first (16 cows) and second (8 cows) measurement periods, 21 records could be used to evaluate the PHDMI prediction equation for hay rations. Three records were discarded due to unrealistic or incomplete behavioral records related to the RumiWatch system or its application. As expected, hay DMI in the barn was considerably underestimated by the equations developed to estimate PHDMI, as indicated by the mean estimated intake (10.3 kg DM/d with WSB3 to 16.4 kg DM/d with the S5 equation) and the local shift (−6.99 for WSB3 to −2.60 for the S4 equation). The average intake rate of 48.2 (±8.9 SD) g DM hay/min was even higher (*P* < 0.001) than the intake rate of fresh herbage in the barn.

Figure 1 presents the box plots and median hay DMI of cows with and without RumiWatch halter. For the same 8 cows, hay DMI in measurement wk 1 (with RumiWatch halter) and in measurement wk 2 (without halter) was the same (*P* = 0.28, 95% CI for the difference between the 2 groups: −0.52 to 0.18 kg/d). Additionally, although the statistical power was low, the average hay DMI recorded during the second measurement week for 16 cows—8 of which were equipped with the halter and 8 without— was also the same (*P* = 0.70, 95% CI for the difference between the 2 groups: −2.02 to 2.92 kg DM hay/d). Wearing the RumiWatch halter does not seem to affect hay DMI in dairy cows. To avoid reducing intake, it is important that the halter is correctly fitted to the animal's head. A description of the correct fitting can be found in Rombach et al. (2018).

**Figure 1:**
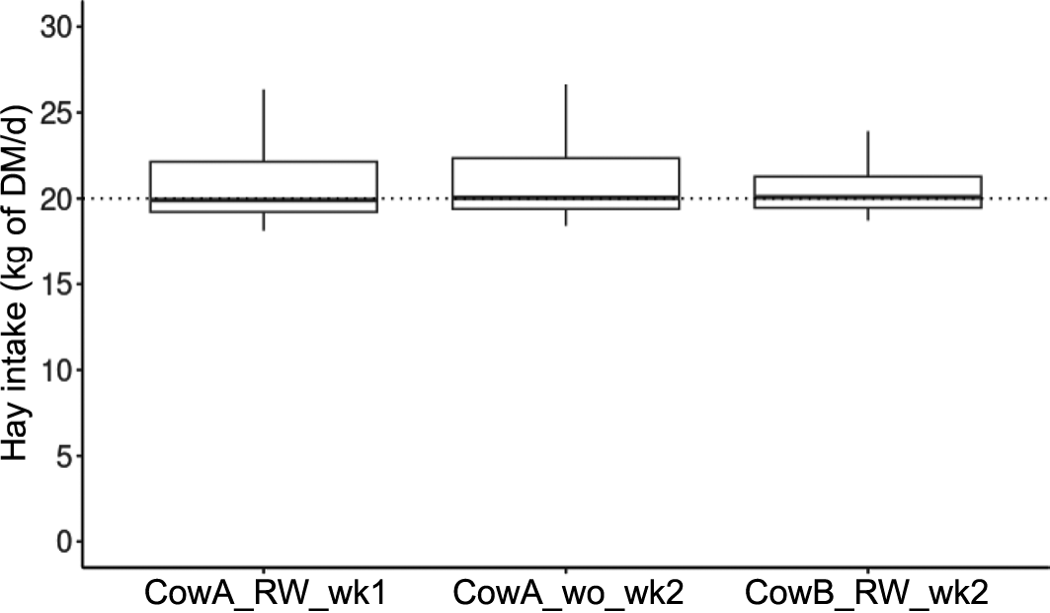
Hay intake (kg DM/d) of cows with or without the RumiWatch halter. CowA_RW_wk1 = 8 cows wearing a RumiWatch halter in the first measurement week. CowA_wo_wk2 = the same 8 cows as CowA_RW_wk1 without a RumiWatch halter in the second measurement week. CowB_RW_wk2 = 8 cows wearing a RumiWatch halter in the second measurement week. Boxes represent the interquartile range (IQR); horizontal lines mark the median; whiskers extend to the most extreme values within 1.5 × IQR; the dotted line indicates a DMI of 20 kg/d.
